# Uncemented total hip arthroplasty in chronic hemodialysis patients: 20 hips reviewed after 2–13 years

**DOI:** 10.3109/17453671003628749

**Published:** 2010-04-06

**Authors:** Wei-Chun Li, Chun-Hsiung Shih, Steve W Ueng, Hsin-Nung Shih, Mel S Lee, Pang-Hsin Hsieh

**Affiliations:** ^1^Department of Orthopedics, Chang Gung Memorial HospitalTaoyuan; ^2^College of Medicine, Chang Gung University; ^3^Department of Orthopedics, Chun Shan Hospital, TaipeiTaiwan

## Abstract

**Background and purpose** Whether or not uncemented total hip arthroplasty (THA) can achieve durable fixation of implants to bone in patients on chronic hemodialysis is unknown. We analyzed the 2–13-year clinical outcomes of cementless THA in patients with end-stage renal diseases who were maintained on long-term hemodialysis.

**Patients and methods** We reviewed the outcome of 23 consecutive uncemented THAs undertaken between 1993 and 2004, in patients with chronic renal failure who had been on long-term hemodialysis (2–18 years). 1 patient died and 2 patients were lost to follow-up within 2 years, leaving 20 hips (20 patients, median age 66 (38–81) years at the time of THA, 11 females) that were reviewed at median 7 (2–13) years postoperatively.

**Results** Radiographic bone-ingrowth fixation of the components was found in 19 patients. 1 patient had aseptic loosening requiring revision surgery. The median d'Aubigne and Postel score was 10 (8–14) preoperatively and 15 (12–18) at final review. No prosthetic infections were found in any of the patients.

**Interpretation** Uncemented THA shows promising medium-term results in patients receiving long-term hemodialysis.

## Introduction

Although uncemented total hip arthroplasty (THA) has gained increasing popularity for a variety of hip diseases, it has not been widely adopted in dialyzed patients (Naito et al. 1994, Toomey and Toomey 1998, Sakalkale et al. 1999). Orthopedic surgeons have been concerned that poor bone stock associated with chronic renal failure would make it less possible to obtain secure and reproducible initial press-fitting of an uncemented device for bony ingrowth and maintain bone-ingrowth stability in the presence of ongoing osteopenia from long-term hemodialysis. However, there has been 1 report using uncemented extensively porous-coated implants in patients on hemodialysis, with good outcome (Nagoya et al. 2005).

We assessed whether uncemented THA with circumferential proximal coating of the femoral component is a viable option for dialysis patients. We report the medium-term clinical and radiographical results in a series of patients on long-term hemodialysis for chronic renal failure, in which a variety of uncemented porous-coated THA components were used.

## Patients and methods

Between 1993 and 2004, 30 consecutive primary THAs were performed on 25 patients with dialysis-dependent renal failure. All patients were maintained on hemodialysis, with treatments provided on a thrice-weekly schedule. During this period, an uncemented procedure was commonly used at our institution. In 2 hips (2 patients), however, a cemented prosthesis was chosen because of the surgeon's concern about the bone quality. 1 patient died (1 hip) and 2 patients (2 hips) were lost to follow-up within 2 years after surgery. For patients who had THA on both hips, we included only the first hip from each patient in order to avoid potential bias from within-individual correlations (Bryant et al. 2006), leaving 20 hips for review at 2–13 years after surgery (Table).

**Table:** Characteristics of the 20 dialysis patients who underwent THA (20 hips)

Age (years) **^a^**	66 (38–81)
Male/female	9/11
Diagnosis	
Osteoarthritis	11
Osteonecrosis	8
Rheumatoid arthritis	1
Duration of hemodialysis (years) ^**a**^	6 (2–18)
Type of prosthesis	
Howmedica	3
Wright	10
Zimmer	5
Corin	2
**^a^** median (range).	
Howmedica: Porous-coated anatomic; PCA (Howmedica, Rutherford, NJ).	
Wright: Perfecta hip system (Wright Medical Technology, Arlington, TN).	
Zimmer: Versys hip system (Zimmer, Warsaw, IN).	
Corin: C-Fit uncemented arthroplasty (Corin Medical Ltd., Cirencester, UK).	

The surgery was carried out under general anesthesia using an anterolateral approach. 4 types of THA implants were used. All of them had circumferential plasma-spray or fiber-mesh coating on the proximal third of the femoral stem, as well as on the acetabular cup. We did not use any extensively coated femoral component. Fixation of the cementless THA components was achieved primarily by press-fit technique. Augmented screw insertion for supplemental acetabular cup fixation was not routine, but was used when necessary as judged by the treating surgeon. There was no need to shift a planned cementless THA to a cemented construct because of inadequate fixation.

Each patient received hemodialysis within 24 h before THA and was not dialyzed again for at least 24 h after surgery. The dose of anticoagulant was kept at a minimum to avoid bleeding from the operative site. Antibiotic prophylaxis was given routinely for 3 days; weight bearing was increased as tolerated, with the patient using crutches or a walker in the first 3 months.

**Figure 1.**
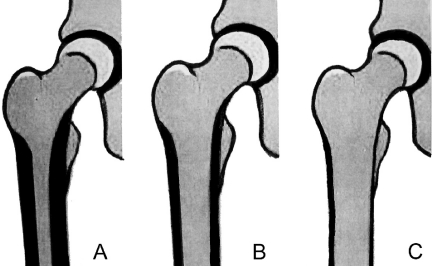
Dorr femoral bone classification. Type A: narrow canal with thick cortical walls (champagne flute canal). Type B: moderate cortical walls. Type C: wide canal with thin cortical walls (stove-pipe canal).

The patients were evaluated clinically and radiographically at 6 weeks, 3 months, 6 months, 1 year, and annually thereafter. The clinical outcome was assessed using the hip score by d'Aubigne and Postel (1954). Preoperative radiographs were categorized according to bone stock types using the Dorr cortical bone classification (Figure 1) (Dorr et al. 1990). Radiographs taken at the latest follow-up were assessed for fixation status of the cementless components. Cup loosening was defined as a change in the cup angle of > 3°, migration of > 5 mm (Hodgkinson et al. 1988), or a complete radiolucent line of > 1 mm in the three zones of DeLee and Charnley (1976). Stability of the stem was assessed by the criteria of Engh et al. (1990) and was classified as having bone ingrowth, being stable fibrous fixation, or being unstable. Polyethylene wear was documented by assessing the eccentricity of the femoral head on the postoperative radiographs using a custom-made computer-aided digitizer (Shih et al. 1997). Osteolysis was defined as a lytic lesion of more than 5 mm in its largest diameter that was not seen on the initial postoperative radiographs (Hsieh et al. 2005).

### Statistics

Nonparametric Mann-Whitney U test was used for comparisons of functional scores before surgery and at the final follow-up. The life table method was used to calculate the survival rate of the prosthesis.

## Results

### Patients with < 2 years of follow-up

2 patients were lost to follow-up within 1 year after surgery and thus no radiographic evaluation was available. 1 patient died in the second year after surgery due to complications that were secondary to end-stage renal disease and not related to the surgery. Radiographs taken 1 year after surgery showed well-fixed components without any evidence of loosening.

### Radiographic results

#### Acetabular side.

**Figure 2.**
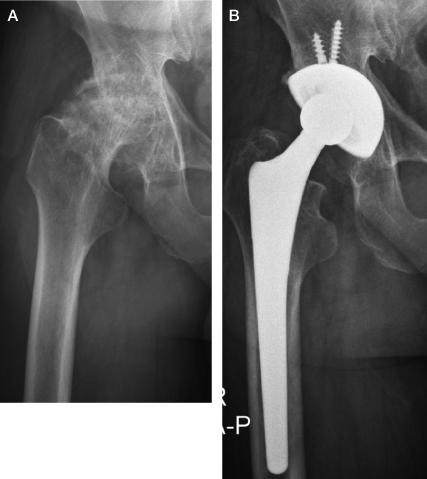
The right hip of a 52-year-old man who was treated with hemodialysis for 12 years. A. Preoperatively, advanced osteoarthritis with a Dorr type B femoral canal. B. 11 years postoperatively, with a well-fixed prosthesis (Perfecta hip system; Wright Medical Technology).

Bone-ingrowth fixation was identified in 19 patients (Figure 2A and B). 1 cup was revised 8 years after the primary THA because of loosening. Measurable linear wear was seen in 4 hips but none had eccentricity of the femoral head of > 2 mm. Cavitary osteolysis was observed in 3 of these 4 hips.

#### Femoral side.

**Figure 3.**
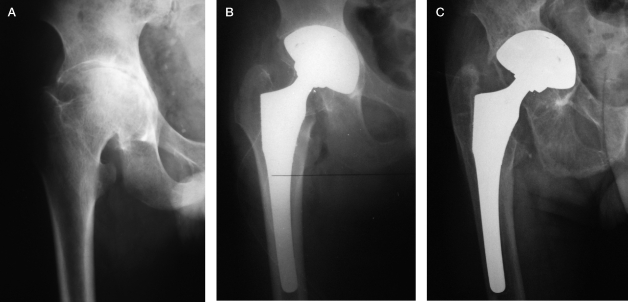
The right hip of a 78-year-old man who had undergone hemodialysis for 13 years. A. Preoperatively, osteoarthritic changes with a Dorr type B femoral canal. B. Immediately after surgery (Porous-coated anatomic; PCA; Howmedica). C. 8 years after THA, showing loosening with migration of the components.

The distribution of Dorr cortical bone types was 3 type A, 12 type B, and 5 type C. Postoperative radiographs demonstrated that adequate canal filling had been achieved in all hips. Based on the classification by Engh et al. (1990), bone-ingrowth stability was obtained in all but 1 hip at the latest follow-up. 1 patient (Dorr type B) who had loosening of the acetabular cup also had a loose femoral stem, presenting radiographically with progressive subsidence and varus malalignment. The stem, together with the cup, was revised 8 years after the index THA (Figure 3A–C).

### Clinical results

There was substantial improvement in postoperative pain relief and joint motion. Walking ability, however, was not considerably improved after THA. At the final follow-up, 11 patients walked normally without a limp or a support, 6 patients could walk for 6 blocks with a cane, and 3 patients were limited to indoor activities with a walker. The median D'Aubigne and Postel score was 10 (8–14) preoperatively and 15 (12–18) at final review. The median scores in the 3 domains improved: pain, from 3 to 6 (p < 0.001); motion, from 3 to 5 (p < 0.001); and walking ability, from 4 to 5 (p = 0.09). The cumulative survival rate was 100% at 5 years and 85% (95% CI, 31–98) at 10 years, with revision for any reason as the end point.

### Complications

In 1 hip, both components were exchanged due to aseptic loosening. 1 patient had fracture of the greater trochanter at the time of THA. Although nonunion was noted at follow-up, no additional surgery was required because of minimal symptoms. 1 patient suffered from peptic ulcer bleeding 3 days after surgery, which was solved with endoscopic local treatment and medication. There was no prosthetic infection, dislocation, neurovascular injury, symptomatic thromboembolic disease, or wound complication.

## Discussion

Patients with chronic renal failure who are on long-term hemodialysis are often not considered for THA with bone-ingrowth fixation because of poor bone quality. Thus, cemented components have often been used in previous studies. Naito et al. (1994) found that 4 of 4 cemented Charnley-Muller prostheses in patients with hemodialysis failed at 7 years, as compared to 7 cases of aseptic loosening in 16 non-uremic patients at 8 years. In a study of 24 hip arthroplasties in dialysis-dependent patients, Toomey and Toomey (1998) reported that 8 of 15 primary cemented stems and 6 of 15 primary cemented cups failed at 8 and 12 years, respectively. Sakalkale et al. (1999) reported that 1 of 7 cemented implants failed because of loosening at an average of 3 years after THA surgery. Although results from the literature are mixed, none of the published studies have shown a satisfactory outcome for chronic hemodialysis patients who had cemented THA.

In our study, we sought to answer the question of whether cementless THA can give durable fixation of implants to bone in patients on long-term hemodialysis. We observed bone-ingrowth fixation in most patients during medium-term follow-up. Whereas we sometimes observed incomplete radiolucent lines around the cementless cups, expanding radiolucency and loosening with migration were found in only 1 hip.

Our results are consistent with a previous report by Nagoya et al. (2005) in which no loosening was found in 11 extensively coated hip implants at follow-up after a median period of 8 (3–13) years. These data suggest that bone ingrowth surfaces can produce adequate fixation of total hip components in patients on long-term dialysis at mid-term follow-up. Although the numbers of patients in our study and that by Nagoya et al. (2005) were small, the results appear to be superior to those recorded for cemented technique in this patient population.

Our study differs from that of Nagoya et al. (2005) in that although we used 4 types of uncemented hip implants, none of them were extensively coated components. We were impressed by the fact that durable fixation could be achieved by the proximally coated stems. It may be argued that since different implants were involved, the study lacked uniformity. An alternative viewpoint is that reliable stability from solid bony ingrowth can be obtained with a variety of cementless implant designs.

The factors that lead to early loosening of THA in dialyzed patients remain unclear. Amyloid deposition, commonly seen in uremic patients who maintain on long-term hemodialysis, is thought to contribute to early arthroplasty failure in these patients. Umeda et al. (1998) found amyloid deposits in all specimens obtained from 4 failed bipolar hip arthroplasties in hemodialysis patients. In a study of 5 uremic patients undergoing surgery for failed primary hip arthroplasties, Crawford and Athanasou (1998) found amyloid deposition in the newly formed connective tissue membrane surrounding the implant components, as well as in the pseudocapsule. However, the data currently available cannot explain a causal relationship between amyloid deposition and early loosening.

Although uncemented THA in uremic patients on hemodialysis provides reliable osseous integration, the overall functional improvement is often limited by other factors. We observed pain relief and increased joint motion in our patients, but walking ability was similar to that before surgery. Only 11 of the 20 patients could walk independently. Advanced age, medical infirmity, and vertebral fractures secondary to osteoporosis often limit walking ability in uremic patients on long-term hemodialysis treatment.

There are a number of safety concerns when treating renal failure patients on hemodialysis with joint replacement. Anemia, electrolyte/fluid imbalance, and cardiovascular events commonly associated with chronic renal failure increase the risk of surgical mortality and morbidity. Malnutrition, coagulopathy, and impaired immunity secondary to long-term hemodialysis may lead to a higher rate of wound complications and prosthetic infection. Only a small number of studies have reported on complications in dialysis patients with THA, and with different findings. In a study of 14 patients who had chronic renal failure and who were receiving hemodialysis treatment, Sunday et al. (2002) reported that 4 of the patients died in hospital during the postoperative period following hip or knee replacement. Toomey and Toomey (1998) reported a complicated hospital course in 8 of 24 chronic dialysis patients undergoing hip arthroplasty, including 1 perioperative death. A prosthetic infection rate of up to one-fifth has also been reported in studies ranging in size from 15 to 46 hips (Naito et al. 1994, Lieberman et al. 1995, Sakalkale et al. 1999). On the other hand, no infection or death related to the surgery was seen by Nagoya et al. (2005), which is consistent with our observations. Comparison of results between the different studies is not possible because of the heterogeneity of the patients. Careful perioperative measures, including preoperative consultations with the cardiologist and the nephrologist, planned transfusion and hemodialysis, adjusted use of anticoagulants, and prophylactic antibiotics for 3 days may all contribute to a good outcome. One other possible reason for low complication rate in our study may be that we did not include uremic patients undergoing non-elective arthroplasty surgery for hip fractures, who are known to be associated with frequent complications and a high mortality rate (Karaeminogullari et al. 2007, Blacha et al. 2009).

Our study was retrospective, with a small number of patients. We believe, however, that our study demonstrates that reliable, durable fixation can be achieved with various porous-coated acetabular and femoral implants in chronic renal failure patients on long-term hemodialysis. The results are similar to, or even better, than those reported earlier on cemented THA in this patient group.
